# *CFG* and the New Year

**DOI:** 10.1002/cfg.68

**Published:** 2001-02


					Editorial
CFG and the new year
This New Year, the (R)rst of the New Millennium,
sees an important landmark in the development of
Comparative and Functional Genomics (CFG). CFG
is no longer a specialised section of Yeast, but is
now a journal in its own right (or is that write?).
Yeast subscribers should not fear, they will continue
to receive complimentary copies of CFG throughout
2001. What it does mean is that CFG will receive its
own citation index and the correct abbreviation for
the journal, when citing its articles, is Comp. Funct.
Genom.
Other (R)rsts in this issue include the (R)rst short
communication' to appear in the journal (in fact,
from my own group!). Authors who wish to adopt
this format should follow the guidance in CFG's
Notes for Contributors'. Two new members of the
Editorial Board join us with the New Year. Takuji
Sasaki (Rice Genome Research Program, Japan)
and Ed Coe (University of Missouri, USA) join
Mike Gale's team. They will cover, respectively, rice
and grasses, and maize and related crops.
This issue has a major Meetings Feature on the
second Agricultural Microbes Genome Meeting
(AMG2) in San Diego. The next Issues will feature
two major model organisms, Arabidopsis thaliana
and Schizosaccharomyces pombe, the genome
sequences of which have recently been completed.
Ju
Erg Ba
Ehler (Sanger Centre, UK) has been recruited
to the Board to help with the anticipated increase in
submissions on the functional genomics of the
(R)ssion yeast. Thus, all seems set for a truly
groundbreaking year for CFG and I wish all of
our readers every success in their research during
2001.
Steve Oliver
Comparative and Functional Genomics
Comp Funct Genom 2001; 2: 3.
Copyright # 2001 John Wiley & Sons, Ltd.

				

